# Can Deep Learning Hit a Moving Target? A Scoping Review of Its Role to Study Neurological Disorders in Children

**DOI:** 10.3389/fncom.2021.670489

**Published:** 2021-05-05

**Authors:** Saman Sargolzaei

**Affiliations:** Department of Engineering, College of Engineering and Natural Sciences, University of Tennessee at Martin, Martin, TN, United States

**Keywords:** ADHD, ASD, cerebral palsy, concussion, deep learning, epilepsy, network neuroscience, neurological disorders

## Abstract

Neurological disorders dramatically impact patients of any age population, their families, and societies. Pediatrics are among vulnerable age populations who differently experience the devastating consequences of neurological conditions, such as attention-deficit hyperactivity disorders (ADHD), autism spectrum disorders (ASD), cerebral palsy, concussion, and epilepsy. System-level understanding of these neurological disorders, particularly from the brain networks' dynamic perspective, has led to the significant trend of recent scientific investigations. While a dramatic maturation in the network science application domain is evident, leading to a better understanding of neurological disorders, such rapid utilization for studying pediatric neurological disorders falls behind that of the adult population. Aside from the specific technological needs and constraints in studying neurological disorders in children, the concept of development introduces uncertainty and further complexity topping the existing neurologically driven processes caused by disorders. To unravel these complexities, indebted to the availability of high-dimensional data and computing capabilities, approaches based on machine learning have rapidly emerged a new trend to understand pathways better, accurately diagnose, and better manage the disorders. Deep learning has recently gained an ever-increasing role in the era of health and medical investigations. Thanks to its relatively more minor dependency on feature exploration and engineering, deep learning may overcome the challenges mentioned earlier in studying neurological disorders in children. The current scoping review aims to explore challenges concerning pediatric brain development studies under the constraints of neurological disorders and offer an insight into the potential role of deep learning methodology on such a task with varying and uncertain nature. Along with pinpointing recent advancements, possible research directions are highlighted where deep learning approaches can assist in computationally targeting neurological disorder-related processes and translating them into windows of opportunities for interventions in diagnosis, treatment, and management of neurological disorders in children.

## 1. Background and History

The brain, as the body commander in chief, evolves by passing through multiple developmental and maturation stages, from the neurogenesis (neuron production stage) and migration (neuron translocation to the neocortex stage) to the differentiation (neurons integration in specialized neural networks by forming axonal connections) (Stiles and Jernigan, 2010). Before transition into the postnatal phase, a massive systematic synaptic exuberance and pruning occurs. After birth, axonal myelination (the process of sheath formation) comes into play that significantly improves axonal integrity and conductance. In support of emerging brain connectivity, synaptic exuberance and pruning continue to occur in parallel with myelination. Tackling neurological disorders by studying the brain structure and function has long been in the neuroscientific research spotlight.

In 1906, an exciting milestone was set for the history of modern neuroscience when the Noble Prize of physiology and medicine was shared between two people, let us indeed reemphasize, between two opponent theories, proposed by Camillo Golgi (1843–1926) and Santiago Ramon y Cajal (1852–1934) (Swanson et al., 2007). Their opposition is rooted in their opinions of how neurons, as the critical units of nervous systems, intercommunicate. Golgi's reticular theory formulated the nervous system as a continuous organization, wherein Cajal's neuron doctrine expressed it as a contiguous organization. While scholarly activities have been on the rise more in favor of the contiguous organization, a growing belief in networks' role has persistently emerged in studying the brain under normal and pathology. The emergence of brain connectivity networks is an essential aspect of brain plasticity, also known as neuroplasticity, a brain adaptation process from experience and learning. During development, brain plasticity rises as it is exposed to environmental events (Rosenzweig and Bennett, 1996). Neuroimaging studies have confirmed the dynamic evolution of these cortical networks through use-induced plasticity to learn and improve functions (Hua and Smith, 2004).

Our current understanding of the connectivity role is the direct consequence of advancements in two main inter-related domains, hardware and computational algorithms. The breakthrough of network-level studies of brain activities was expedited by a series of technology inventions that began by Electroencephalography (EEG) (Britton et al., 2016) and continued with Magnetoencephalography (MEG) (Cohen, 1968), X-ray, Computed Tomography (CT), and Positron Emission Tomography (PET) in the 70's (Raichle, 2009). In the late 70s, Magnetic Resonance Imaging (MRI) was introduced, further boosting brain-related discoveries. The expedition offered by the MRI technology itself was obscured until the advent of different MRI sequences. The MRI sequence refers to a particular harmony set between radio-frequency pulses and magnetic gradients, leading to capturing a specific perspective from the tissue appearance. In 1990 (Ogawa et al., 1990), oxygen consumption and supply to cerebral regions was, for the first time, considered as an endogenous contrast agent for recording brain functional activities. The method, called Blood-Oxygen-Level-Dependant (BOLD) functional MRI, has since been a dominant MRI method of choice in resting-state functional MRI (rs-fMRI) task-based functional MRI, both based on the regional association of brain activity with oxygen supply and consumption. Shifting the focus from functional imaging, The knowledge of brain microstructure diffusion properties has driven diffusion-weighted imaging (Basser et al., 1994) to streamline structural imaging. In parallel to hardware-related technological advancements, computational and visualization power has immensely matured following the development of computing backbones such as C/C++ and python programming languages and the ease of virtual memorization and parallel processing. The development of tools like AFNI (Cox, 1996), FreeSurfer (Fischl, 2012), FSL (Jenkinson et al., 2012), and SPM (Penny et al., 2011) has complemented these inter-related efforts. The rising investment in portraying the brain and its role in diseases and disorders is reflected amid initiatives such as the Brain Initiative or the International Brain Initiative.

**Figure 1:**
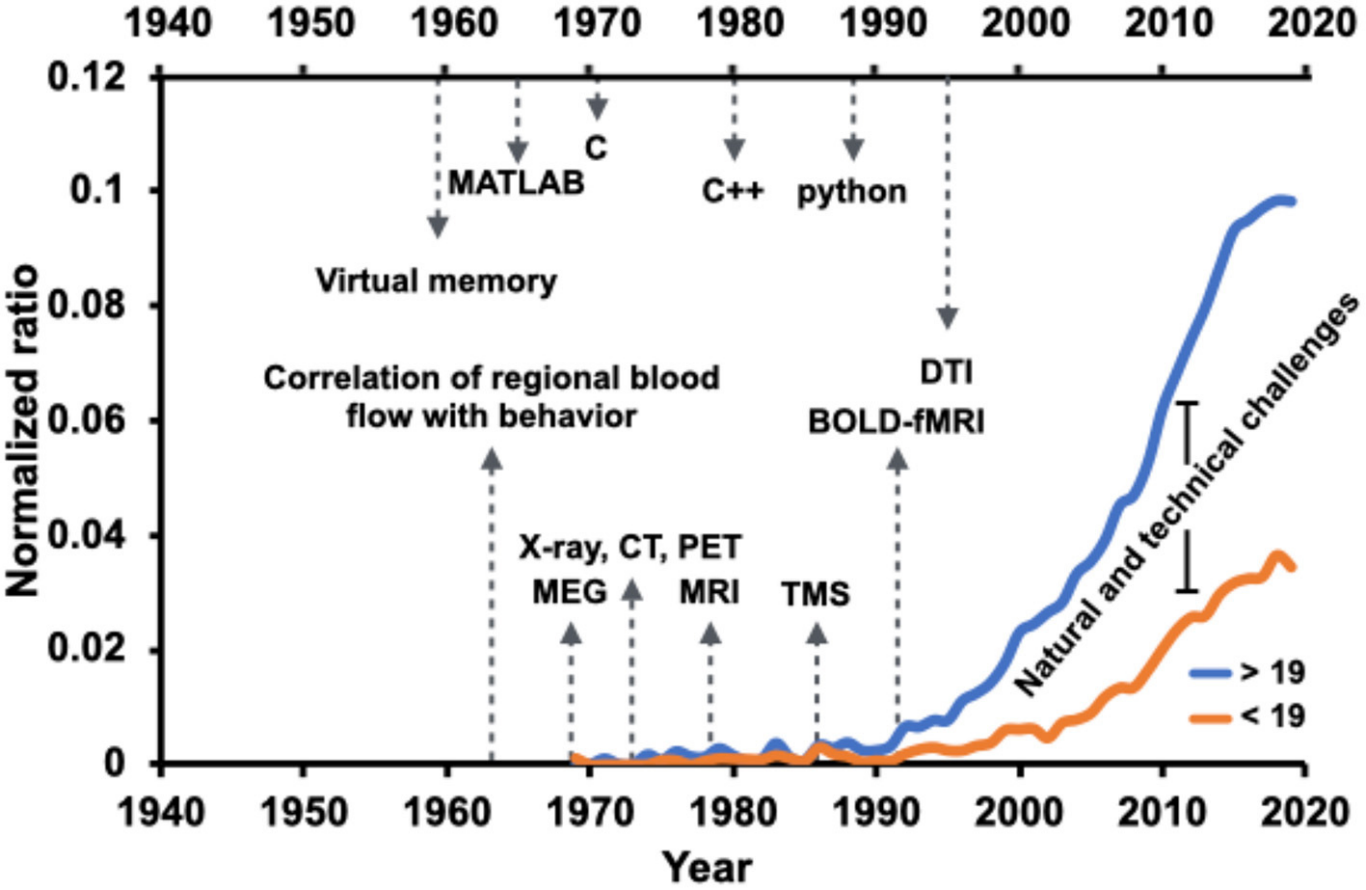
Brain network-related scholarly activities in the pediatric population (younger than 19 years old of age) have paced at a slower rate compared to the adult population (more aged than 19 years old of age), a possible reason being the natural and technical challenges. Normalized ratio of the PubMed-indexed full text articles including [(brain) AND (network)] term, normalized by the total number of publications found by [(brain)] keyword, overlaid with advancement milestones in the field of technology and computation. The annual ratio is visualized for two age groups, younger and older than 19 years old.

The field has been immensely grown since Golgi and Ramon y Cajal's neuroanatomical work that emphasized the role of the functional interplay among regional brain structures. Nevertheless, the growth has happened at a lower rate within discoveries related to the knowledge we have about children's brain networks. Figure 1 captures a synopsis of scholarly activities within the “brain network” domain over the years highlighted with milestones of technology advancements. Contrasted by the age of 19 years old, the graphs show the annual number of full-text articles found in the PubMed search engine with [(brain) AND (network)] term, normalized by the total number of publications found by [(brain)] keyword. From the early 1970s, there has been a steady increase in brain research's scholarly activities, particularly under the umbrella of the brain network. However, the increase in trends shows a slower rate for studies in 19 years old and younger age population.

## 2. Challenge and Prospect

**Figure 2:**
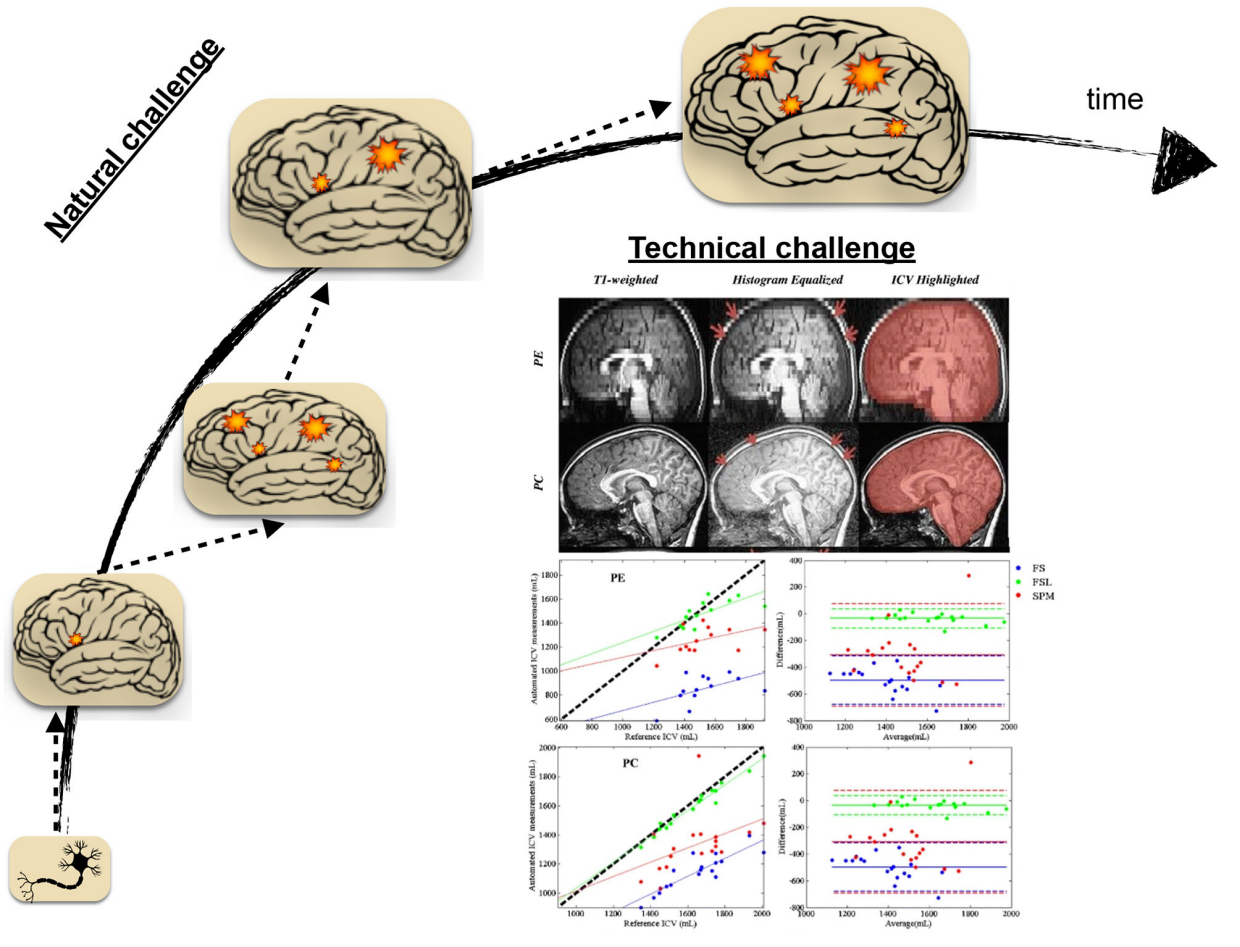
Natural and technical challenges in the face of studying neurological disorders in the pediatric population. Over time, brain development involves many ongoing processes from the cellular to the network level. In the presence of disease or disorder, The ensemble of normal brain development processes is occluded by dynamic processes led by disease or condition, making the clinical studies naturally challenging in pediatric age groups. Technical challenges, including procedural difficulties and data processing methodological limitations, are also surrounding the brain's studies in the pediatric population. The presented technical problem here is reproduced with permission from Figures 2, 4 of Sargolzaei et al. (2015) for the task of intracranial volume estimation (ICV) using automated tools in two pediatric age group of human subjects (PE, Pediatric Epilepsy; PC, Pediatric Control).

Natural and technical challenges (Figure 2) could be deemed for the slower trend of scholarly activities related to pediatric brain studies. The natural, or to better frame it, the inherent difficulty in studying the developmental brain is the development factor's attachment. The development of a complex biological entity, such as the human brain, entails significant synergies among phased development stages, from the genetic blueprint to the formation of brain structure on the foundation of millions of brain cells, including neuronal and glial cells (Gibb and Kovalchuk, 2018). It becomes even further intricate when adding the element of functional network formation to the developmental stages. Brain circuitry emergence is the result of brain cell interactions over time. It is a set of highly dynamic processes, including iterative formation and elimination of synapses and stabilization of relevant synaptic connections to mature functional brain activity in conjunction with brain structure (Kuczmarski, 2002; Hua and Smith, 2004). Aside from the genetic blueprint, it is recognized to depend upon other factors, including nutrition, environmental exposures, and experiences such as interactions with people (Robinson et al., 2017).

The amendment of disease-driven processes to the ensemble of normal brain development processes introduces further uncertainty and complexity, particularly for studies concerned with accurate delineation of normal development operations from those operations driven by pathology. The natural challenge is to an extent that cautious needs to be practiced before generalizing findings in neuroimaging studies of children and adolescents (Santosh, 2000). While neuroimaging, especially non-invasive ones, has become increasingly popular in the era of brain scientific investigation (Morita et al., 2016), its use is confined by issues such as non-compliance (Schlund et al., 2011; Raschle et al., 2012; Thieba et al., 2018). Non-compliance refers to the interruption or failure of the experiment, often due to subject movement or failure to abide by instruction due to anxiety. The issue arises even further for the clinical neuroimaging studies in children and adolescents to the extent that it necessitates proper considerations and study protocols (Greene et al., 2016).

The unique challenges of clinical studies currently extend beyond non-compliance to procedural difficulties, technical obstacles, and data processing methodological limitations. In this regard, The development and utilization of age-appropriate equipment can further streamline clinical studies in children and adolescents. Along with procedural and technical considerations, specific adjustments, age-dependent protocol developments, and analytical fine-tuning are essential for correct implications of neuroimaging studies in the pediatric population (Sargolzaei et al., 2015).

Figure 2 highlights an example of the technical considerations that must be practiced when the neuroimaging study population is pediatrics. It features the task of automatic intracranial volume (ICV) estimation in brain research. ICV estimation is a critically required task in neuroimaging studies. While the task may be fulfilled by manual inspection and landmarks identification, it is a tedious and laborious process. Automatic estimation involves the utilization of neuroimaging software packages and the implementation of a proper ICV estimation routine. As it is apparent from the shown figure, different software packages led into different ICV estimation when contrasted to the reference estimation of such quantity under different condition (control sample vs. pediatric patients with epilepsy)(Sargolzaei et al., 2015). The study emphasizes methodological challenges for pediatric neuroimaging studies, pointing out the necessity of guided decision-making in selecting developed tools under different circumstances.

To overcome the above-summarized challenges, indebted to the availability of high-dimensional data and increased computing capacities, approaches based on graph theory and machine learning have rapidly emerged a new trend to understand pathways better, accurately diagnose, and adequately manage the disorders. Deep learning has recently gained an ever-increasing role in the era of health and medical investigations under the umbrella of machine learning-based solutions to studies of neurological disorders. For the field of neurological disorders in children, our prospect is at the conjunction of applied deep learning engaged with graph theory on a personalized scale.

## 3. Deep Learning, Something New or the New Face of the Old

Granting machines the gift of intelligence is an ever-increasing appetite for humankind, and the learning skill is standing at the forefront of this intellectual gift. Through decades of artificial intelligence field evolution, machines learned through human-generated rules and accurately engineered features. This approach, conveniently referred to as the classical approach, has phenomenally succeeded in multiple application domains, yet extracting an optimized set of features and rules is not always cumbersome. Further tasks, such as recognition, which is intuitive for humans, remained a challenge for machines to learn via classical approaches that rely upon the existence of high-level abstract features (Goodfellow et al., 2016).

The deep learning approach's neurobiological spirit roots back in experimental studies (Hubel and Wiesel, 1959; Métin and Frost, 1989; Roe et al., 1992) discovering the mutually critical roles of intracortical circuit specifications and inputs to such circuits in the learning process of brain neural networks. Learning about the experience-dependant plasticity of the brain (Simons and Land, 1987; Kirkwood et al., 1995; Crist et al., 2001; Trachtenberg et al., 2002) inspires the methodological possibility of letting the machine also acquire knowledge by experience. Thorough experience-dependent knowledge acquisition involves exposure to a vast amount of such experiences, accompanied by corresponding outcomes, and the capacity to automatically form an intra-circuitry that maps these experiences to their corresponding outcome. Translating these requirements to artificial neural networks' jargon entails a specialized architecture, ultra-large datasets, and sophisticated linear and non-linear training algorithms, formulating the deep learning approach to the machine learning task. The specialized network architecture allows sequential processing of the supplied raw data units through multiple layers without the need for human-based feature design and engineering.

**Figure 3:**
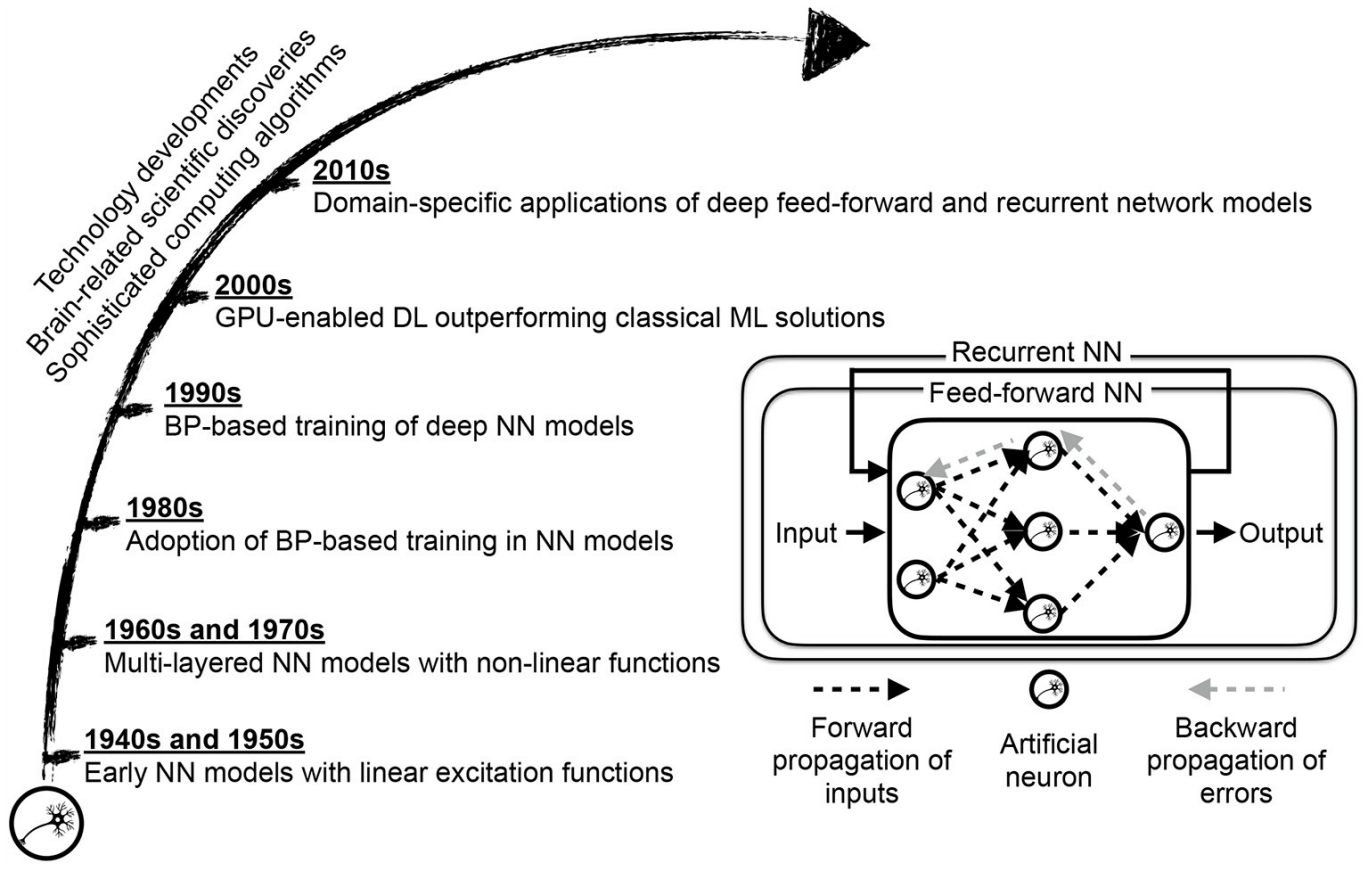
Deep learning (DL) approach maturation motivated by scientific discoveries of brain structure and function and enabled by computing technology and algorithms' immense progress. The core of a neural network (NN) model is an artificial neuron, resembling a biological neuron in a limited way, evaluating the collective input signals followed by conditional excitement. While early citations of NN models, as a linear regression variation, dates back to the 1800s, NN-based learning was not widespread until the development of sophisticated training algorithms based on back-propagation of error signals, and gradient descent optimization (Schmidhuber, 2015). The transition from classical NN-based learning, where the network examines extracted features from data to deep NN-based learning, where the network scans raw data representation, requires equipping NN with self-exploring capacity through multiple layers of abstraction. The graphical processing unit (GPU) based computing partially enabled such a self-exploratory multi-layered representation learning to the extent that deep learning becomes the choice method in numerous domain-specific applications (LeCun et al., 2015).

The deep learning approach (Figure 3) lets the network itself find an optimized allocation of credits to basic units (neurons) of these layers in sketching the desired outcome (LeCun et al., 2015; Schmidhuber, 2015). The network depth offers a way to break down a complex raw input into simplified units of information, mainly through convolution and sampling processes, and collectively map the input to the provided network output. Self-exploration mapping is the backbone of architectures such as deep convolutional neural networks (CNN) and recurrent neural networks (RNN), with the latter one empowering the learning of sequential data such as text and speech. Alongside other application sectors, healthcare has reported breakthroughs achieved with deep learning adoption in neuroimaging, genetics, oncology, radiation therapy, and drug discovery, to name a few (Wang et al., 2018, 2020; Boldrini et al., 2019; David et al., 2019; Serag et al., 2019; Tang et al., 2019; Zhu et al., 2019; Chen et al., 2020; Zhang et al., 2020).

## 4. Deep Learning and Neurological Disorders in Children

To review the scope of deep learning application in diagnosis, treatment and management of neurological disorders in children, we surveyed existing peer-reviewed literature. The search was performed using PubMed (https://pubmed.ncbi.nlm.nih.gov), and IEEE Xplore (https://ieeexplore.ieee.org/Xplore/home.jsp). The search queries were set to [(deep learning) AND ((children) OR (child) OR (pediatric) OR (paediatric))] for PubMed search, and to [((“All Metadata”:Deep learning) AND ((“All Metadata”:children) OR (“All Metadata”:child) OR (“All Metadata”:pediatric)))] for IEEE Xplore search. Setting the inclusion criteria to peer-reviewed journal articles reporting the deep learning approach utilization in studying, diagnosing, or managing neurological disorders in the pediatric (up to 19 years of age) population led to a total of 22 records. Included articles were examined to retrieve the following information, study neurological condition (*Condition*), study population age range (*Age range*), study goal (*Goal*), modality of the used data (*Modality*), used deep learning model (*Model*), and backbone implementation environment (*Environment*). Table 1 summarizes included studies.

**Table 1:** A summary of found peer-reviewed journal articles utilizing deep learning to study, manage, diagnose, and prognosis of neurological conditions in pediatric (18 years or younger) populations.

Condition^*^	**References**	Age range^**^	**Goal**	Modality^***^	Model^****^	Environment^*****^
ADHD	Muñoz-Organero et al., 2018	[6–15]ye	Distinguishing medicated from non-medicated activity pattern	Tri-axial accelerometer	CNN	MATLAB
	Amado-Caballero et al., 2020	[6–15]ye	Automatic diagnosis of combined type ADHD	Activity recordings	CNN	MATLAB
	Muñoz-Organero et al., 2019	[6–16]ye	Assessment of movement patterns	Tri-axial accelerometer	LSTM-based RNN	
	Chen et al., 2019	[9–12]ye	Detecting spatiotemporal anomalies	EEG	CNN	
ASD	Bahado-Singh et al., 2019b	[24–79]ho	Identification of early biomarkers	Leucocyte DNA	Deep neural network	R h2o
	Hazlett et al., 2017	[6–24]mo	Prediction of autism diagnosis	MRI	Deep neural network	MATLAB
	Eni et al., 2020	[23–73]mo	Prediction of ASD severity	Speech	CNN	
	Aghdam et al., 2018	[5–10]ye	Classification of ASD from typically development control	rs-fMRI and sMRI	DBN	Theano
	Ghafouri-Fard et al., 2019	[6–14]ye	ASD status detection	Genomic data	Deep neural network	Keras
CP	Bahado-Singh et al., 2019a	[24–79]ho	CP prediction	Leucocyte DNA	Deep neural network	R h2o
Concussion	Boshra et al., 2019	[15–20]yy	Classification of concussed from control	EEG/ERP	CNN	Tensorflow v1.8.0
CHD brain dysmaturation	Ceschin et al., 2018	[35–45]we	Dysplastic cerebelli detection	MRI	CNN	Theano
Epilepsy	O'Shea et al., 2020	[39–42]we gestational	Seizure detection	EEG	CNN	
	Ansari et al., 2019	Neonates	Classifying epileptic from non-epileptic seizures	EEG	CNN with RF	MATLAB
	Bernardo et al., 2018	[2–60]mo	Detection of fast-ripples	EEG	CNN	Theano
	Daoud and Bayoumi, 2019	[3–15]ye	Early detection of pre-ictal state	EEG	CNN	
	Lin et al., 2020	[7–16]ye	Distinguishing patients without ED from controls	EEG	CNN	
	Lee et al., 2020	[4–16]ye	Tract classification for preoperative analysis	MRI-DWI	CNN	PyTorch 0.2
	Xu et al., 2019	[6–17]ye	Preoperative detection of axonal pathways	MRI-DWI	CNN	PyTorch 0.2
Brain tumor	Quon et al., 2020	92mo median	Posterior fossa tumor detection	MRI	CNN-ResNeXt	Keras
Schizophrenia	Aristizabal et al., 2020	[9–16]ye	Risk assessment	EEG	RNN	Keras
TSC	Sánchez Fernández et al., 2020	[5–16]ye	Cortical tubers detection	MRI	CNN	TensorFlow

* *Condition is the neurological condition studied in each reference (ADHD, attention-deficit/hyperactivity disorder; ASD, autism spectrum disorder; CP, cerebral palsy; CHD, congenital heart disease; TSC, Tuberous sclerosis complex).*

** *Age range data is estimated and rounded from the given information for each reference (ye, years; ho, hours; mo, months; we, weeks).*

*** *Modality refers to the data types used for learning (EEG, electroencephalograph, DNA, Deoxyribonucleic acid; MRI, magnetic resonance imaging; rs-fMRI, resting-state functional MRI; ERP, event-related potential; DWI, diffusion-weighted imaging).*

**** *Model describes the core model utilized for deep learning given provided information in each reference (CNN, convolutional neural network; LSTM, long short-term memory; RNN, recurrent neural network; DBN, deep belief network; RF, random forest).*

***** *Environment is where the deep learner being implemented in each referenced study (MATLAB by MathWorks, R interface for H2O package for large-scale machine learning, Theano, python library supporting large-scale multidimensional computations, Keras, A python deep learning API, TensorFlow, An open-source machine learning platform, PyTorch, an open-source machine learning framework*.

Perceived from Table 1, The deep learning architectures, in particular convolutional neural networks, have reported promises in a wide variety of applications, including automatic diagnosis, biomarker identification, early detection, within-condition type classification, and risk assessment for a variety of neurological conditions in children. The survey reveals the applicability potential of deep learning solutions beyond neuroimaging modalities, such as EEG and MRI, extending its use to other information sources such as speech and activity-based modalities within the pediatric clinical population. The latter inference from the survey is of utmost importance due to the challenges discussed before regarding methodological constraints of studies in pediatrics. While python-based libraries are the dominant library of choice reported in the current studies sample, MATLAB and R language libraries for large-scale machine learning implementations are also cited.

While deep learning solutions are relatively free of conventional feature engineering requirements, they are entangled with their choice of architecture and learning parameters, enforcing the need for careful implementation and selection of such internal architecture and its relevant parameters. The consequences of varying architecture are reported to have an impact on the deep learner performance for the use of convolutional neural network (Xu et al., 2019) and deep belief network (Aghdam et al., 2018). Therefore, the inclusion of benchmark routines, mainly reporting different architectures for optimum architecture selection, can enhance study findings.

Our survey search has also identified a rising interest in using deep learners to overcome inherent challenges, low tissue contrast, and dynamic appearance variation for brain imaging tasks in infants (Guo et al., 2014; Zhang et al., 2015; Kawahara et al., 2017; Mostapha and Styner, 2019; Sun et al., 2019) and fetus (Girault et al., 2019; Dou et al., 2020). A common goal for most surveyed articles is to conduct more extensive validation studies that involve multi-site investigations to make deep-learning-based computational tools more suitable as the non-invasive and inexpensive real-time clinical tool of choice.

## 5. Viewpoints and Conclusion

The current scoping review concentrated the scope on the rising use of deep learning to study, diagnose, and prognosis for children's neurological disorders. The hallmark of neurological disorders is the presence of brain dysfunction. Presentation of behavioral and psycho-behavioral symptoms is a consequence of such dysfunction. Neuroimaging studies have confirmed the dynamic evolution of these functional cortical networks through use-induced plasticity. In a similar context, neurological disorders have been shown to leave the pathophysiological signature on brain networks. Network neuroscience, in this regard, has significantly matured and been extensively utilized in studying neurological disorders; however, the pace of brain network understanding and its role in driving neurological conditions presentation in children falls behind that of the older age population.

We discussed inherent challenges in the face of studying neurological disorders in the pediatric population, contrasting it to the task of hitting a moving target. Brain plasticity is a complex and heterogeneous phenomenon, reflected as a multi-faceted maturation. The pace of brain development process variation occurs at a relatively higher rate in the early stages. Overlaying such a dynamic with uncertainties imposed by neurological disease and disorders turns the task of studying pediatric neurological studies into a difficult one. Inspired by the way our brain sees and learns the world, computational advancement gave birth to a new horizon, called deep learning, promising revolutionary insights into the field of medicine and biology.

Our scoping review of the current state of deep learning utilization in children's neurological disorders has identified a rising interest from scientific investigators for deep learning in various classification tasks related to children's neurological disorders. Data volume, along with, to a lesser extent, data quality, remains among major barriers to incorporate deep learning for studying neurological disorders (Miotto et al., 2018; Valliani et al., 2019). The emergence of deep learning will further continue in the era of pediatric clinical studies because of its lesser reliance upon the existence of engineered features.

Aiming at the development of the generalized deep learner to the level of clinical efficacy requires taking a steeper, and yet clear, road. It involves conducting more extensive multi-sites validation studies and performing benchmark evaluations of deep learning architectures to intensify their utilization further. Studying less utilized architectures such as recurrent neural networks and auto-encoders to learn the joint temporal dynamics of the disease and development in one shot will form a valuable complement to the existing efforts. Legal, privacy, and ethical challenges, with respect to the use of deep learners in healthcare (Miotto et al., 2018; Valliani et al., 2019), remain at place with greater emphasis for the vulnerable populations such as pediatrics. Efforts such as distributed deep learning schemes (Zeng et al., 2018; Remedios et al., 2019) can partially help resolving such dilemma. While deep learning is reported to outperform traditional machine learning algorithms, joined solutions need to be evaluated in cases where deep learners may supplement the established solutions.

Like hitting a moving target, studying neurological disorders in the developing brain is a cumbersome task. However, as tracking a moving target before hitting it can increase success, performing multi time-points longitudinal studies in the pediatric population can improve our chances of understanding and defeating the disease. Therefore, longitudinal modeling and analysis of children's neurological disorders using deep learning architectures can unravel windows of opportunities to hit the moving target.

## Author Contributions

SS contributed to all stages of study, contributed to manuscript revision, read, and approved the submitted version.

## Conflict of Interest

The author declares that the research was conducted in the absence of any commercial or financial relationships that could be construed as a potential conflict of interest.
